# Supporting good practice in the provision of services to people with comorbid mental health and alcohol and other drug problems in Australia: describing key elements of good service models

**DOI:** 10.1186/1472-6963-10-325

**Published:** 2010-12-03

**Authors:** Monika Merkes, Virginia Lewis, Rachel Canaway

**Affiliations:** 1 Australian Institute for Primary Care & Ageing, Faculty of Health Sciences, La Trobe University, Melbourne, Australia; 2 School of Psychology and Psychiatry, Faculty of Medicine, Nursing and Health Sciences, Monash University, Melbourne, Australia

## Abstract

**Background:**

The co-occurrence of mental illness and substance use problems (referred to as "comorbidity" in this paper) is common, and is often reported by service providers as the expectation rather than the exception. Despite this, many different treatment service models are being used in the alcohol and other drugs (AOD) and mental health (MH) sectors to treat this complex client group. While there is abundant literature in the area of comorbidity treatment, no agreed overarching framework to describe the range of service delivery models is apparent internationally or at the national level. The aims of the current research were to identify and describe elements of good practice in current service models of treatment of comorbidity in Australia. The focus of the research was on models of service delivery. The research did not aim to measure the client outcomes achieved by individual treatment services, but sought to identify elements of good practice in services.

**Methods:**

Australian treatment services were identified to take part in the study through a process of expert consultation. The intent was to look for similarities in the delivery models being implemented across a diverse set of services that were perceived to be providing good quality treatment for people with comorbidity problems.

**Results:**

A survey was designed based on a concept map of service delivery devised from a literature review. Seventeen Australian treatment services participated in the survey, which explored the context in which services operate, inputs such as organisational philosophy and service structure, policies and procedures that guide the way in which treatment is delivered by the service, practices that reflect the way treatment is provided to clients, and client impacts.

**Conclusions:**

The treatment of people with comorbidity of mental health and substance use disorders presents complex problems that require strong but flexible service models. While the treatment services included in this study reflected the diversity of settings and approaches described in the literature, the research found that they shared a range of common characteristics. These referred to: service linkages; workforce; policies, procedures and practices; and treatment.

## Background

The co-occurrence of mental illness and substance use problems (referred to as "comorbidity" in this paper) is common, and is often reported by service providers as the expectation rather than the exception [1]. Despite this, many different treatment service models are being used to treat this complex client group in the alcohol and other drugs (AOD) and mental health (MH) sectors. Despite a range of government and agency initiatives to minimize barriers to treatment and build strong partnerships between drug treatment and mental health services [2-6], recent findings suggested that people with a history of illicit drug use and co-occurring anxiety or depression are still not well serviced by AOD and MH services in Australia [7,8].

In Australia, comorbidity treatment or care may be administered by various levels of government and non-government organizations. Models of service delivery may vary in these different sectors, reflecting their level of access to resources and their individual funding, management, policy, and service development structures. Federal and state policy environments also affect the local approach to managing service delivery for comorbidity. As AOD and MH services in Australia are generally administered and funded separately, a clear policy framework for the development of well-defined treatment models has been lacking.

While co-occurring mental health and substance use disorders have attracted increasing attention from various levels of government and independent bodies, comorbidity is recognized in the literature as an area that still lacks a cohesive or comprehensive framework from which to address the issues of prevention, awareness, screening, assessment, treatment and ongoing support for those with co-occurring disorders.

Consistent with overseas experience [9], in Australia there is no consensus in the definition of comorbidity. Definitions are narrowly to broadly conceived [10,11]. Narrow definitions commonly limit comorbidity to the co-occurrence of severe mental illness (e.g. psychotic disorders) with concurrent substance use [12]. Broad definitions can encapsulate all mental health disorders and any level and combination of substance use problems. The diversity of conceptual frameworks for comorbidity presents a barrier to comparability of research and has implications for the delivery of treatment programs [9]. Regardless of the specific definition used, the co-occurrence of mental health and substance use disorders is generally associated with complex mental, physical and psychosocial problems and needs, the expression of which may vary across different treatment settings [9,13].

Descriptions of models of service delivery for comorbidity in the literature generally fall under the headings of sequential, parallel, or integrated:

• Sequential (or "serial") models are those in which treatment is provided by different clinicians in different settings. One disorder is treated in isolation, followed by treatment for the second disorder.

• Parallel models are those in which treatment is provided concurrently by different clinicians in different settings. There may or may not be communication between providers.

• Integrated models may be implemented at the service/system, single-sector or client/program level

◦ Service/system level integrated models are distinguished by coordination, collaboration, or linkages between independent service providers, particularly MH and AOD providers, to facilitate coordinated treatment for the individual.

◦ In single-sector integrated models, either the AOD or MH sector act as the primary provider of integrated treatment for individuals with comorbidity. In these models treatment may be limited to a particular type or level of comorbidity.

◦ Client/program level integrated models reflect the coordinated treatment of both mental health and substance use disorders by a single treatment agency or clinician. Either individual clinicians are trained across MH and AOD disciplines or several clinicians work in multidisciplinary teams.

In addition to models of service delivery, the literature reflects some common principles that may be incorporated into these models. The concept of a "no wrong door" approach expresses the aim that all presenting clients will be provided with appropriate treatment services or referral, consistent with their treatment needs, regardless of where they enter the treatment system [14]. The principle of a "flexible fit" reflects the concept that the treatment model or path is not pre-determined and the same for all clients. The particular needs and circumstances of each client are considered in combination with the available treatment options and an individualized treatment plan is developed.

Abundant literature is available about comorbidity, but few controlled studies compare service system models or approaches to provide evidence of their effectiveness or to explain how they work [e.g. [15-17]]. Further, no agreed overarching framework to describe the range of service delivery models is apparent internationally or at the national level. Despite limited evidence, there is broad (but not total) support for integrated models of service delivery [16,18,19], but little information about the prerequisites or conditions that may be required to support delivery of this kind of model.

As indicated above, the complexities associated with defining comorbidity and the methodological challenges for research in the field have resulted in little agreement on what constitutes good practice in the delivery of treatment services to people with comorbid disorders. The current research to explore this issue was funded by the Australian Government Department of Health and Ageing (DoHA) under the umbrella of the National Comorbidity Initiative (NCI).

The aims of the research were to identify and describe elements of good practice in current service models of treatment of comorbidity in Australia. The focus of the research was on models of service delivery. The research did not aim to measure the client outcomes achieved by individual treatment services, but sought to identify elements of good practice.

A literature review conducted as part of the broader research program [11,20] focused on peer reviewed papers pertaining to co-occurring mental health and substance use issues; specifically, treatment models or frameworks, service delivery and implementation, and service improvement and management. CINAHL, Medline, Embase, Informit, PsychINFO and the Cochrane Library were searched for English languages articles published between 2000 and 2008. Earlier literature was included if pertinent reference was made to it in the 2000-2008 literature. A search of relevant Australian and international government and agency information clearinghouses, networks and databases provided additional contextual, program-specific and policy-related materials including Australian Government policies such as the National Drug Strategy [21] and the National Mental Health Strategy [22].

**Figure 1:**
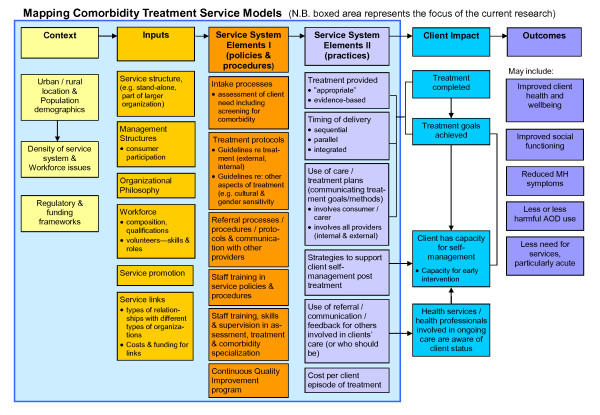
**Mapping Comorbidity Treatment Service Models**. Figure 1 comprises the service model map for the study. The service model map provided the framework for the research.

Informed by the literature and consultation with 10 experts in the field, commonly described key components of service models used in the treatment of comorbidity were identified. Using these elements as the foundation, and drawing on program logic modeling [23-25] a general overarching logic map of service delivery was devised (Figure 1). The map summarizes the common elements that emerge from descriptions of different service models and good practice. It represents an hypothesized map onto which different service models could be superimposed in order to consider the way in which different elements may combine to reflect service models that support good practice.

The map describes the elements of comorbidity treatment services, from the contextual factors that affect service models through to the intended outcomes for clients.

• Context: These are factors related to geographic location (e.g. rurality), density of the service system (i.e. availability of comorbidity and other relevant services, such as primary care services, and links between these services), and regulatory frameworks (e.g. state regulatory requirements or other issues with funding bodies that impact on the service) within which services operate.

• Inputs: These reflect the way the service is set up, including its structure (e.g. whether the service is part of a government organization), the workforce it has, and the way in which it promotes its service.

• Service System elements - Policies and Procedures: These elements reflect formal statements that guide the way in which treatment is delivered by the service.

• Service System elements - Practices: These aspects reflect the way treatment is provided to clients.

• Client impacts: These elements are the consequences of delivery of services from a client perspective, and include completion of treatment as well as the way in which the service ensures that care (by self and others) will continue after the client leaves the service.

• Client Outcomes: These are the intended consequences of delivery of good quality treatment by the service. It was beyond the scope of this study to measure the outcomes achieved by the participating services. This represents a limitation to the current study, and highlights an area for future research.

The research aimed to identify ways in which the selected services were structured and operated within the overarching framework, and to consider commonality within and between different kinds of services.

## Methods

Seventeen Australian treatment services were identified to take part in the study. The services were identified through a consultative process involving a range of key stakeholders, including 10 key informants who also provided advice and insights with regard to the literature review [20]. Key informants were nominated by their peers as Australian experts in at least one of the following areas: comorbidity; mental health; substance misuse; rural/metropolitan health care settings; and/or service delivery design. A key criterion for nomination of services to participate in the research was that it was perceived to achieve good outcomes for clients with comorbidity issues.

The list of services to be included was finalised in agreement with the funder, with the final list designed to include services that represented different target client age groups (i.e. adult, child and adolescent), rural and metropolitan locations, residential and non-residential services, and different states/territories. The intent was to look for similarities in the service delivery models being implemented across a diverse set of services that were perceived to be providing good quality treatment for people with comorbidity problems.

Based on the domains and sub-domains of the comorbidity service logic map (Figure 1), a survey was devised to explore how each of the 17 nominated 'good practice' services operated within each of the domains. After testing the survey tool with two services, the survey was conducted online between December 2008 and February 2009. It included 84 questions designed to elicit quantitative and qualitative data, and was administered in two parts. Each of the participating treatment services identified one or two staff to complete the survey, with one survey per service. Respondents were clinicians and senior managers. Further information about the survey is available elsewhere [26].

Ethics approval for the study was received from the La Trobe University Human Ethics Committee, and consent received from respondents at the commencement of the online survey.

Analysis of the data was completed with the assistance of SPSS (V17) and occurred at service level and across services. Due to the small number and diversity of treatment services evaluated, only descriptive statistics were used in the analysis of the quantitative data. The qualitative data obtained from the survey provided additional detailed information about the 17 treatment services.

## Results - Service characteristics

The structure of this section follows the domains and sub-domains outlined in the Comorbidity Treatment Service Models map (see Figure 1) and refers to Table 1 and 2 which summarize the organization, client and staffing characteristics of the services.

**Table 1:** Organization and client service characteristics

ID	Service type and structure			Client treatment service type					Links/partnerships with other orgs. (No.)	Service System elements: policies and procedures				Treatment type		
	Type*	Size**	Ind/Sub ***	Community based out-patient	Residential					Screened for co-morbidity at intake	Explicit treatment protocols exist	Routine outcome measures used	Treatment plans given	Serial/sequential	Parallel	Integrated
													
					Res	No. of beds****	Average occupancy (%)	Average stay (days)								
**A**	AOD	Sml	Ind	✗	✔	B	67%		14	✔ (All)	✗	✔	✔ (All)	✗	✗	✔
**B**	AOD	Sml	Ind	✔	✗				8	✔(Most)	✗	✔	✔(Some)	✗	✔	✔
**C**	AOD	Sml	Ind	✗	✔	B	80%	70	38	✔ (All)	✔	✗	✔ (All)	✗	✔	✗
**D**	AOD	Sml	Sub	✔	✗				12	✔(Most)	✗	✔	✗	✗	✗	✔
**E**	AOD	Sml	Sub	✔	✗				27	✔ (All)	✗		✔ (All)	✔	✔	✔^
**F**	AOD	Sml	Sub	✔	✔	A	83%	65	13	✔ (All)	✗	✗	✔ (All)	✗	✗	✔
**G**	AOD	Sml	Sub	✔	✗				26	✗	✗	✗	✗	✗	✔	✗
**H**	AOD	Sml	Sub	✗	✔	B	70%	90	10	✔ (All)	✔	✔	✔ (All)	✗	✗	✔
**I**	combined	Sml	Ind	✔	✗				22	✔ (All)	✔	✗	✔ (All)	✗	✗	✔
**J**	combined	Sml	Sub	✗	✗^^					✔(Some)	✔	✔	✔ (All)	✔	✗	✔
**K**	combined	Sml	Sub	✔	✗				30	✗	✗	✗	✔(Most)	✔	✗	✗
**L**	combined	Sml	Sub	✔	✗				14	✔ (All)	✗	✔	✔ (Most)	✗	✗	✔
**M**	AOD	Med	Ind	✔	✔	C		33	22	✔(Most)	✔	✔	✔ (All)	✗	✗	✔
**N**	AOD	Med	Sub	✔	✔	A	80%	6	20	✔(Most)	✔	✔	✔ (All)	✗	✗	✔
**O**	combined	Med	Ind	✔	✔	C	95%	290	42	✔ (All)	✔	✔	✔ (All)	✔	✗	✔
**P**	AOD	Lrg	Sub	✔	✔	B	88%	27	14	✔(Most)	✔	✔	✔.(All)	✗	✔	✗
**Q**	combined	Lrg	Sub	✔	✔	**C**	91%	9	15	✔ (All)	✔	✔	✔ (All)	✔	✔	✔

**Totals**				13	9				19.2 average	15	9	11	15	5	6	13

* AOD = Alcohol & Other Drugs; combined = AOD and mental health service (combined)

** Service size is determined by number of employed clinicians and other professional staff; small (Sml): 2 to 20, medium (Med): 27-80, large (Lrg): 124-403

*** Ind = Independent organization. Sub = Subsidiary of a larger organization

**** To avoid identification of services, the services have been grouped: up to 19 beds (A), 20-39 beds (B), and 40-70 beds (C)

^ This service indicated it would use treatment models appropriate to individual client needs and the expertise of clients

^^ This service was part of a larger organization. This particular service reported that it provided neither out-patient or residential care

Note: empty cells--not applicable or information was not provided

**Table 2:** Staffing characteristics of organizations

ID	Total No. Staff (EFT) excl. volunteers	Total No. Clinical Staff (EFT)	Average years worked in service	% completed minimum required qualifications	Trained to identify comorbid problems	Professional development is a requirement	Continuous Quality Improvement program in place
**A**	15		4	74%	✔	✔	✔
**B**	25.6	10	2	100%	✗	✔	✔
**C**	29		4	75%	✔	✔	✔
**D**	4		1	100%	✔	✔	✔
**E**	6.5		5	62%	✔	✔	✔
**F**	9	3	2	100%	✔	✔	✗
**G**	14	14	4	95%	✔	✔	✗
**H**	14		5	100%	✔	✔	✔
**I**	21.2	12	2	100%	✔	✔	✔
**J**	2		15	100%	✔	✔	✔
**K**	2.4		4	60%	✔	✔	✔
**L**	5		5	100%	✔	✔	✗
**M**	96		2	90%	✔	✔	✔
**N**	69		6	98%	✔	✔	✔
**O**	73.3	29	5	98%	✔	✔	✔
**P**	143.3	116	2	70%	✔	✗	✔
**Q**	423		2	95%	✔	✔	✔

**Totals**			4.1 years average	89% average	16	16	14

Note: empty cells--information was not provided

### Context

#### Service types and settings

While services were deliberately chosen to reflect a diversity of client groups and locations, their nomination as models of 'good practice' was of primary consideration. Participating services represent a range of contexts and different structures. The treatment services were funded through a combination of different state/territory and/or Australian Government funding arrangements with no single policy or program supplying funding for their complete service provision. Eight of the services reported that underfunding impacted negatively on their capacity to deliver services.

One service reported that it serviced rural and remote communities predominantly; six identified their catchment area as a whole state/territory (and one reported a catchment of more than one state), and five reported a "whole region". Only three reported their catchment at the local government area.

### Inputs

#### Service structure and promotion

Fifteen of the treatment services identified as being part of the non-government or private-not-for profit sector, and two as government sector organizations. Eleven reported being subsidiaries of larger organizations, while six were stand-alone or independent services. As indicated in Table 1 the providers described themselves as AOD services (n = 11) or as combined AOD and MH services (n = 6). Three of the latter group also provided other services, such as welfare or sexual health. Of the 17 services, two catered specifically for Indigenous clients and five for adolescents/young people only. Nine services provided a residential program, and 13 provided a community-based/outpatient service. One service described itself as part of a larger organization and reported that it offered neither residential nor community-based outpatient services (Service J, Table 1). This service rarely provides direct services to clients: services are primarily provided to regional Community Mental Health (CMH), AOD and Psychiatric Disability Rehabilitation and Support (PDRS) agencies and clinicians. All providers reported actively promoting their service, particularly through relevant networks, flyers, information stalls at special events, and through their websites.

#### Organizational philosophy

Respondents were asked to "describe the philosophy or guiding principles of your program". Most (n = 11) reported a "harm reduction" or "harm minimization" approach, and ten reported working in a way that is "holistic", flexible, and/or client-centered. One small service reported abstinence as their guiding principle and another reported theirs to be "Christian values".

When providers were asked whether they used a framework or model to classify comorbidity, three said they did, 11 said they did not, and three indicated they did not understand the question. One framework was defined by reference to the state health department's minimum data set and its approach to recording comorbidity; one was described in terms of DSM IV criteria; and a third related to the "level of disability or disturbance measured with the degree of addiction and dependence". Comments from other respondents suggested that at least three services were operating within informal frameworks or models of comorbidity similar to the three described.

#### Workforce

When the 'good practice' services are categorized according to the number of clinicians and other professional staff employed (total Equivalent Full Time positions), into small (n = 2-20), medium (n = 27-80) and large (n = 124-403), most (n = 12) fall into the small category.

All services listed the minimum qualifications they required for staff and reported that most staff had completed or were completing the required qualifications (see Table 2). Overall, there were no substantial differences in the required minimum qualification for AOD workers and counselors in AOD and combined services.

The mean number of years that staff had worked for the services ranged from three years for clinical staff to five years for staff in managerial roles. The means were similar across AOD compared with combined service types and the size of categories.

Nine services reported roles for volunteers, with the time contribution made by people in these roles ranging from less than one to 400 hours per week per service (the latter primarily staffing fund-raising opportunity/thrift shops).

#### Service links

Providers were asked about the relationships they had with other services or organizations (see Table 1). They reported a range of links including networking, coordinating, cooperating, and collaborating relationships. Medium-sized services reported the largest number of links with other services (mean number of links = 28), followed by small (19) and large organizations (15). The most frequent collaborating relationships were those with AOD and MH treatment services, followed by GPs (family physicians), housing services, and the criminal justice system. The most effective links, according to respondents, were those between AOD and MH services.

### Service system elements: Policies and procedures

#### Intake

Sixteen services described intake processes that included an initial screening interview or assessment process conducted by qualified staff to determine whether and when an individual would be accepted into their service (see Table 1). All but two services screened for comorbidity, although only nine services said they screen all clients for comorbidity. Six services reported using validated screening tools, five reported using "purpose-built screening tools", and four said they used a combination. Several respondents spoke of inadequate screening tools for their particular client target groups, and one considered that screening tools were not required because they conducted a comprehensive clinical assessment and screening would be "superfluous".

#### Treatment

Nine services reported that their service had treatment protocols and/or guidelines and manuals (see Table 1). These included guidelines for developing and implementing treatment plans, guidelines around gender matching clients and clinicians, protocols for case management, and promotion of particular evidence-based interventions.

#### Referral

All services reported discharge planning policies and procedures including formal discharge plans (n = 15), as well as letters or discharge summaries sent to referring services, GPs or the courts. Ten services reported that part of their discharge process was to link clients with other relevant services, including "testing" them prior to a client leaving the program. In the case of one service that reported "informal" processes, this included a "discharge pack" with information about how to re-access the service if required.

#### Staff professional development

Sixteen of the 17 services reported that staff are required to undertake continuing professional development (PD) (see Table 2), with seven services reporting that staff received four or five days of professional training in the past 12 months, and nine reporting that staff had received more than five days of PD. Eight services reported funding allocated to PD as a per capita figure ranging from $200 to $1,000 per person per year (mean $590), while four reported spending between two and five per cent of the program budget on PD. All services provide regular supervision to clinical staff, with 13 reporting more than one type of supervision. Small organizations were less likely to provide multiple types of supervision.

Most services (n = 13) reported that staff are trained in the organization's referral procedures. Two services thought they should be, and two said that clinical staff do not need to be trained in referral procedures. With one exception, services reported that some or all of their staff had received training in identification and treatment of clients with comorbid problems during the previous 12 months (see Table 2).

#### Continuous Quality Improvement (CQI)

The majority of services reported having CQI programs in place (n = 14) or being in the process of introducing such a program (n = 1). Ten different programs were nominated. Small services were less likely to participate in CQI programs (see Table 2).

### Service system elements: Practices

#### Treatment type and timing

Most services reported providing integrated treatment (n = 13); fewer reported using parallel treatment (n = 6) and serial/sequential treatment (n = 5) models. Several respondents noted, however, that they may vary the model according to the client's need and/or the clinician providing treatment. Only one AOD service used serial/sequential treatment models, and this was in addition to both parallel and integrated approaches (see Table 1 service E).

The majority of surveyed services reported that all (n = 12), most (n = 2) or some (n = 1) clients had individual treatment plans (n = 15), with two small AOD services not developing individual treatment plans (see Table 1). Fifteen services said clients are always involved in development of treatment plans and carers are sometimes involved, with 14 also indicating that other providers are sometimes involved. Most reported the plans were provided to the client (n = 9) or to the client and carer (n = 6).

#### Referral

Responses to the questions of whether feedback about clients was provided to referral sources reflect the complexity of policies and practice. Six of the services always provide feedback, nine usually do, and the remaining three do not. One service provided two responses in order to indicate that the provision of feedback was related to the type of client; feedback was always provided for clients mandated by the judicial system, but feedback about other clients may or may not be provided. Other respondents provided similar comments to indicate that there were reasons that feedback was not always provided.

#### Client self-management strategies

Thirteen services reported supporting clients in self-management after discharge from the program, including providing home visits, individual counseling, post-withdrawal support groups, playgroup, phone links, employment support and day programs. Three providers said they taught self-care skills as part of treatment, including providing information about services, transport and accommodation, as well as training in relaxation and problem-solving.

#### Cost

The majority of services did not answer the question about average treatment costs. Those who did provided different figures, including cost per bed, cost per enrolment, cost per episode of outreach or care, and cost per clinician. Five services reported that they did not monitor the cost of treatment.

### Client Impact

It was beyond the scope of the current study to link service characteristics to client impact. Future research is needed to explore this.

## Discussion

As expected, the services examined were diverse in terms of their settings, their size, their funding sources, and their structures--there was no dominant model. A range of philosophies or guiding principles was reported, but the most common were "harm reduction" or "harm minimization" and a flexible, client-centered approach.

When the different elements of the comorbidity service models map were explored through a survey of respondents, there were strong areas of commonality across services, despite the differences in their contexts and structures.

Given that the treatment services in this study were selected on the basis of perceived good practice and performance, the identified characteristics of good services are tentative and need to be tested in future research.

The following outlines the main themes of the study, contrasts these with findings of other research, and identifies limitations of the study.

### Complexity

As reported in the literature, the complexities associated with comorbidity and methodological challenges have resulted in little agreement on what constitutes good practice in the delivery of treatment services to people with comorbid disorders. The lack of consensus in regard to the definition of comorbidity and treatment approaches have implications for prevention, awareness, screening, assessment, treatment, and ongoing support for those with co-occurring disorders.

Reflecting the diversity described in the literature, the evaluation found no commonalities in regard to frameworks used to classify comorbidity, and most services did not explicitly use a classificatory model. However, further probing may have revealed the existence of implicit frameworks.

### Common elements of good practice

The development and maintenance of linkages and partnerships with a diverse range of allied services to ensure specialized, coordinated treatment, and continuity of care for clients is generally considered good practice [e.g. [2,27]]. The surveyed services reported a range of links with relevant organizations, including networking, coordinating, cooperating, and collaborating relationships. The strength of links between AOD and MH sectors reported by these services is in contrast to reports in the literature which argues that collaboration is very poor [e.g. [28]], suggesting it is an important element for good practice.

The services selected to participate in the study generally had well-qualified staff and generous provision of supervision and professional training. The overall level of qualified staff and the lack of difference between AOD and combined services in their level of staffing contrasts with the literature, where it is commonly reported that the AOD treatment workforce includes a high number of counseling staff with experience-based rather than formal training [e.g. [29]]. Although no state or national workforce data with which to compare it exists, it appeared that the services all had relatively stable staff.

We found that services had explicit policies and procedures including those related to intake, comorbidity screening, treatment guidelines, referral, discharge planning, and client feedback. While the details of their policies and procedures varied, all services described having clear mechanisms for intake, including comorbidity screening. Most services reported using screening tools, either validated, "purpose-built" or a combination. Approximately half of the services had treatment guidelines, manuals or protocols, and all reported having discharge planning policies and procedures. Furthermore, most of the services reported they had a CQI program in place, although 10 different programs were nominated. CQI programs are thought to bring about substantial and sustained improvement in the quality of care [e.g. [30,31]].

The literature provides little guidance on good practice in regard to feedback to referring professionals. Whether or not feedback to referral sources is provided depends, for example, on program philosophy, privacy policies, and/or professional notions of client confidentiality. It could be argued that a well-functioning partnership between services bridging the AOD and MH fields requires, at minimum, communication and feedback about client diagnosis, treatment(s) provided, and client progress. Such communication mechanisms may require development of and consensus on referral feedback protocols between AOD and MH organizations at a local, regional, or broader level. Our evaluation found that feedback--formal or informal--is commonly provided to referring professionals. In some services, this depends on the type of provider (e.g. GPs and the criminal justice system are most likely to receive feedback) and/or on client consent to share information.

While a preference for integrated treatment compared to parallel or sequential treatment is reported in the literature [16,18,19], in this sample of perceived "good practice services", most AOD and combined services reported providing integrated treatment and, to a lesser degree, parallel treatment. Some combined services also reported providing serial/sequential treatment. The key finding was that, along with all three of the currently described service delivery models being used by participating services, several also commented that they may vary the model according to the client or clinician characteristics. Individual treatment plans were a part of treatment for all but two small AOD services, and all services who used treatment plans reported involving clients in their development. Carers and other providers were also involved by most services.

Some areas for potential improvement in service models emerged from the study, particularly in relation to collection of data that could help to develop this research further. While a majority of services provided an estimate of client treatment completion rates and client outcomes, most did not use validated instruments to evaluate their service efficacy. In addition, many services were not able to provide information about the costs of treatment. This finding is consistent with reports in the literature that the availability of relevant data is scant, variable in quality, and difficult to compare. For example, it has been argued that "few national collections include information about treatment outcomes and it is difficult to comment on the effectiveness of services in terms of client outcomes" [[32], p. 53].

With the service model logic map as a framework within which to represent service models, it would be possible to make a more thorough systematic investigation of the relationship between different elements and client outcomes. In order to do this, there would need to be more consistency in some of the data that services collect.

### Limitations of the study

The current study of comorbidity treatment service models focused on service structures and diagnostic and treatment methods. All 17 services were perceived to be good models of service provision to people with comorbid disorders, but it was not possible to confirm these services did achieve better outcomes for clients than did other services in Australia. It is also worth noting that the study was based on self-report, and it could be argued that respondents were likely to provide the best possible presentation of their service; however, some of the responses that were provided were self-critical, which suggests that at least some respondents endeavored to provide a frank assessment of their service. The small number of services surveyed for this study of service models did not lend itself to analyses based on sub-categories according to different characteristics or elements of the service model. Further, it was not possible to explore how findings from this study might apply to client groups of varying ages and with different conditions.

## Conclusions

The treatment of people with comorbidity of mental health and substance use disorders presents complex problems that require strong but flexible service models. While the treatment services included in this study reflected the diversity of settings and approaches described in the literature, the research found that they shared a range of common characteristics. These common elements or characteristics of good practice relate to: service linkages; workforce; policies, procedures, and practices; and treatment.

In the absence of a consensus on the definition of comorbidity and given the frequency of comorbid problems, a diversity of good practice service models can coexist and provide a 'no wrong door' approach to the treatment of people with comorbid problems.

Using the service model logic map developed for this research it may be possible to test and extend the research further in future, and contribute to a greater understanding of how to achieve the best possible outcomes for clients with comorbidity problems, and to more objectively identify 'best practice' comorbidity services.

## Competing interests

The authors declare that they have no competing interests.

## Authors' contributions

MM developed and implemented the surveys, analyzed the data, and drafted the manuscript. MM and VL developed the service model map. VL contributed to the development of the surveys, analysis of data, and development of the manuscript. RC wrote the literature review element of the project (reported elsewhere) and provided critical input to the manuscript. All authors read and approved the final manuscript.

## Pre-publication history

The pre-publication history for this paper can be accessed here:

http://www.biomedcentral.com/1472-6963/10/325/prepub
